# Supplementary material for: Restraint of Powassan virus replication by TRIM5α facilitates viral avoidance of antiviral immunity
Source: Commun Biol. 2026 Jul 7;9:1419. doi: 10.1038/s42003-026-10613-1 (PMC13720048; doi:10.1038/s42003-026-10613-1)

nature portfolio

Peer Review File

Restraint of Powassan virus replication by TRIM5α facilitates viral avoidance of antiviral immunity

Corresponding Author: Dr Sonja Best

This file contains all reviewer reports in order by version, followed by all author rebuttals in order by version.

Version 0:

Reviewer comments:

Reviewer #1

(Remarks to the Author)
This manuscript presents a rigorous and elegant investigation into the interaction between primate TRIM5α and tick-borne orthoflaviviruses, with a particular focus on Powassan virus (POWV). The authors provide compelling evidence that TRIM5α functions as a host restriction factor against several tick-borne flaviviruses by targeting the viral NS3 protein, and that POWV can evade TRIM5α-mediated restriction through a single amino acid substitution. Intriguingly, the study reveals that such restriction may paradoxically benefit the virus by limiting early innate immune activation, thereby facilitating immune evasion. Overall, this work addresses a significant gap in our understanding of intrinsic immunity to tick-borne flaviviruses and illuminates a novel evolutionary interplay between host restriction factors and viral replication machinery.

My concerns and suggestions are listed below:

1. The cytokine, chemokine, and IFN responses in human MDDCs are only measured at 24 and 48 hpi (Figures 7 and 8). It remains unclear when TRIM5α-mediated restriction is most critical, and whether immune activation is a cause or consequence of elevated viral replication. Including earlier time points (e.g., 6–12 hpi) for IFN-β, CXCL10, and ISG expression would clarify the kinetics of innate immune sensing.
Furthermore, the distinction between TRIM5α's direct antiviral activity and its potential role in modulating innate immune signaling remains ambiguous. Perturbation of IFN signaling—e.g., using IFNAR blockade or JAK inhibitors—could help isolate these effects.

2. The co-immunoprecipitation data (Figure 2H) demonstrate an interaction between TRIM5α and NS3 but lack quantitative assessment of binding affinity or specificity. Biophysical approaches such as surface plasmon resonance (SPR) or biolayer interferometry (BLI) would strengthen claims regarding direct recognition and reveal how V559 or T561 mutations alter interaction kinetics.

3. The relatively modest difference in disease phenotype between WT and mutant POWV in C57BL/6J mice (Figure 6E–H) is not unexpected, given that murine Trim30d does not functionally mimic primate TRIM5α. A human TRIM5α transgenic or knock-in mouse model may more accurately reflect the in vivo relevance of this restriction factor.

4. Current viral growth curves are based solely on infectious virus titers. This metric does not differentiate between viral RNA replication, particle assembly, or release. Inclusion of replicon-based assays or reporter-tagged constructs (e.g., NS3-luciferase fusion) could allow quantitative, time-resolved measurement of intracellular replication.

5. The statement that TRIM5α is "not a primary determinant of neuroinvasion" is plausible but may benefit from more nuanced framing. The absence of NS3 mutations in clinical isolates may reflect purifying selection to avoid excessive innate immune activation, rather than a lack of functional relevance of TRIM5α in the human host.

6. The authors might consider discussing whether POWV could evolve compensatory mutations in other nonstructural proteins (e.g., NS2A, NS5) to mitigate the immunostimulatory consequences of T561M while preserving TRIM5α resistance. This could broaden our understanding of viral adaptation strategies.

Reviewer #2

(Remarks to the Author)
The authors report TRIM5α inhibits Powassan virus replication by degrading the nonstructural proteins NS2B and NS3 encoding the viral protease and RNA helicase and a mutation in NS3 paly a role in this process. The objective of this research is interesting, and the experimental design of the research work is reasonable.
1.Please provide the full name of the proper noun when it first appears.
2.Please divide Table 1 into three tables based on different viruses according to the characteristics of the table.
3.Please further optimize the language expression according to the needs of the writing.
4.Please give a schematic overview of the whole research based on the results.

Version 1:

Reviewer comments:

Reviewer #1

(Remarks to the Author)
The authors have satisfactorily addressed all comments and suggestions raised during the review process. I have no further concerns.

Reviewer #2

(Remarks to the Author)
I concerned has been addressed in revised manuscript.

Open Access This Peer Review File is licensed under a Creative Commons Attribution 4.0 International License, which permits use, sharing, adaptation, distribution and reproduction in any medium or format, as long as you give appropriate credit to the original author(s) and the source, provide a link to the Creative Commons license, and indicate if changes were made.
In cases where reviewers are anonymous, credit should be given to 'Anonymous Referee' and the source.
The images or other third party material in this Peer Review File are included in the article’s Creative Commons license, unless indicated otherwise in a credit line to the material. If material is not included in the article’s Creative Commons license and your intended use is not permitted by statutory regulation or exceeds the permitted use, you will need to obtain permission directly from the copyright holder.
To view a copy of this license, visit https://creativecommons.org/licenses/by/4.0/

COMMSBIO-25-5979-T Response to Reviewers:

Reviewer #1 (Remarks to the Author):

This manuscript presents a rigorous and elegant investigation into the interaction between primate TRIM5α and tick-borne orthoflaviviruses, with a particular focus on Powassan virus (POWV). The authors provide compelling evidence that TRIM5α functions as a host restriction factor against several tick-borne flaviviruses by targeting the viral NS3 protein, and that POWV can evade TRIM5α-mediated restriction through a single amino acid substitution. Intriguingly, the study reveals that such restriction may paradoxically benefit the virus by limiting early innate immune activation, thereby facilitating immune evasion.
Overall, this work addresses a significant gap in our understanding of intrinsic immunity to tick-borne flaviviruses and illuminates a novel evolutionary interplay between host restriction factors and viral replication machinery.

Response: We thank the Reviewer for their very positive comments.

Specific concerns and suggestions:

1. The cytokine, chemokine, and IFN responses in human MDDCs are only measured at 24 and 48 hpi (Figures 7 and 8). It remains unclear when TRIM5α-mediated restriction is most critical, and whether immune activation is a cause or consequence of elevated viral replication. Including earlier time points (e.g., 6–12 hpi) for IFN-β, CXCL10, and ISG expression would clarify the kinetics of innate immune sensing. Furthermore, the distinction between TRIM5α's direct antiviral activity and its potential role in modulating innate immune signaling remains ambiguous. Perturbation of IFN signaling—e.g., using IFNAR blockade or JAK inhibitors—could help isolate these effects.

Response:
We used human monocyte derived dendritic cells (MDDCs) infected with recombinant wild-type (WT) and NS3:T561M Powassan viruses (POWV). At 6, 12 and 24 hours post infection (hpi), expression of *IFNB1* and *CXCL10* mRNA was measured, the latter chosen as the cytokine most responsive to infection. We coupled this analysis with measurement of POWV genomic RNA amplification. Differences between rWT POWV and rPOWV NS3:T561M genomic RNA amplification were not observed at 6 or 12 hpi, and were only observed by 24 hpi. Substantial differences in *IFNB1* and *CXCL10* mRNA were also not observed at the 6 and 12 hpi timepoints. Therefore, TRIM5α restriction and innate immune sensing are both occurring in a similar window, between 12 and 24 hpi of MDDCs. This new work is included in Figure 7 panels D, E, and F.

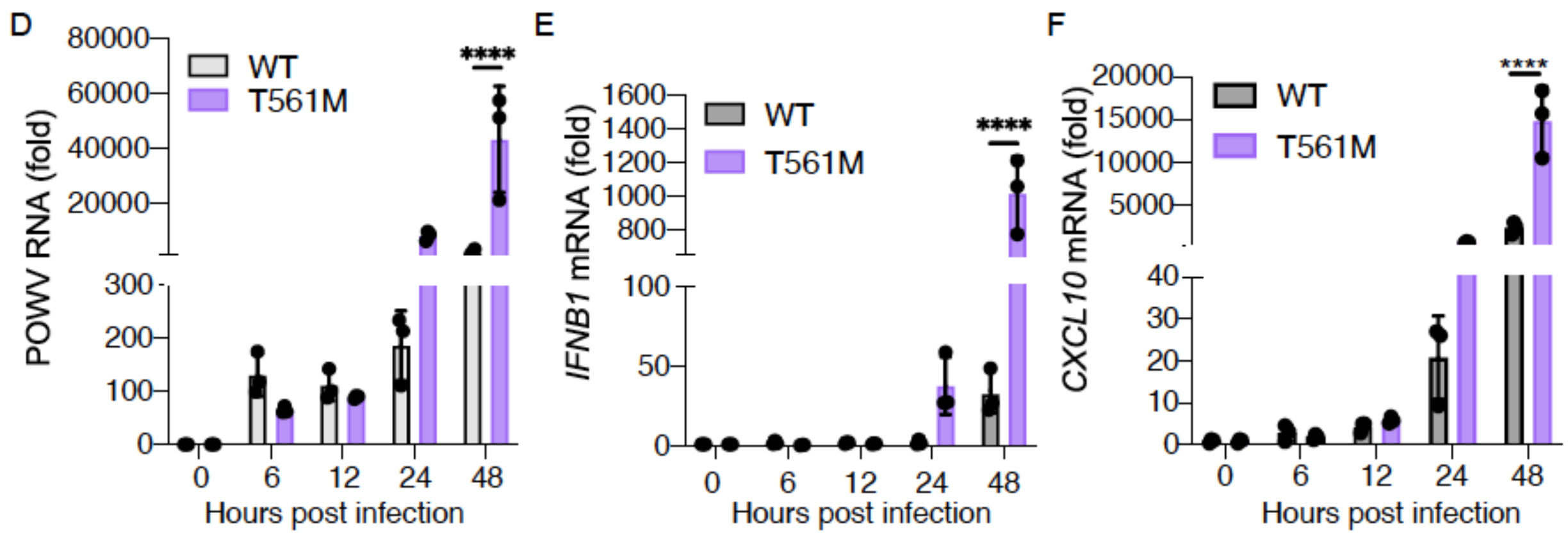

Thank you for the suggestion to use JAK inhibitors. This experiment was particularly informative. We completed these experiments by treating MDDCs infected with rWT POWV or rPOWV NS3:T561M with the JAK inhibitor Ruxolitnib. These experiments were revealing, in that they showed that replication of only TRIM5α-resistant rPOWV NS3:T561M was sensitive to inhibition of JAK-STAT signaling at 48 hpi. Furthermore, also genome replication was significantly increased in RUXO-treated cells, expression of IFNβ protein was significantly suppressed. This new work is included in Figure 8 panels C and D, and strongly supports the conclusion that viral restriction by TRIM5α restrains POWV replication to levels that avoid innate immune activation.

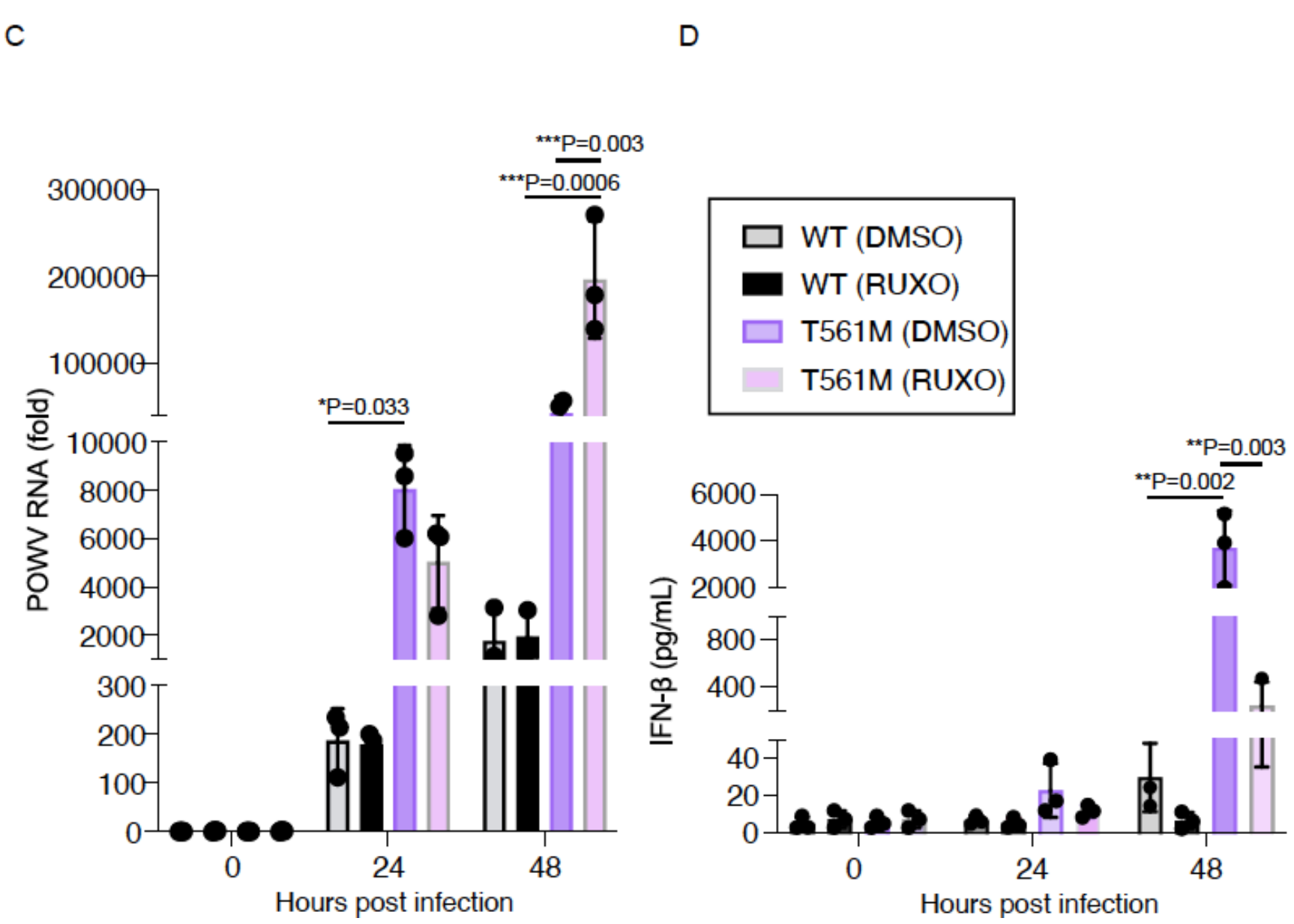

2. The co-immunoprecipitation data (Figure 2H) demonstrate an interaction between TRIM5α and NS3 but lack quantitative assessment of binding affinity or specificity. Biophysical approaches such as surface plasmon resonance (SPR) or biolayer interferometry (BLI) would strengthen claims regarding direct recognition and reveal how V559 or T561 mutations alter interaction kinetics.

Response: Thank you for this suggestion. We appreciate that the precise contribution of the V559 (LGTV) or T651M (POWV) mutation to binding between NS3 and TRIM5α has not been assessed in this manuscript. We have unpublished data that TRIM5α recognizes NS3 in a two-step process, that includes binding to the unlipidated ATG8/LC3B known to decorate orthoflavivirus replication organelles (PMID: 30608919). This first step enables TRIM5α to somehow access NS3 and bind directly. It is possible that the V559 (LGTV) or T651M (POWV) mutation does not directly affect TRIM5α binding affinity, but rather affects the first step in recognition of the unlipidated ATG8. The role of unlipidated ATG8 has been reported to aide in virion assembly (PMID: 30608919). As this is a complicated explanation that takes multiple new experiments to effectively demonstrate, we prefer not to include this data at this time.

3. The relatively modest difference in disease phenotype between WT and mutant POWV in C57BL/6J mice (Figure 6E–H) is not unexpected, given that murine Trim30d does not functionally mimic primate TRIM5α. A human TRIM5α transgenic or knock-in mouse model may more accurately reflect the in vivo relevance of this restriction factor.

Response: We agree that this would be a very interesting model to investigate. However, to our knowledge this transgenic mouse has not yet been generated, and therefore experiments using this model fall outside the scope of the current study.

4. Current viral growth curves are based solely on infectious virus titers. This metric does not differentiate between viral RNA replication, particle assembly, or release. Inclusion of replicon-based assays or reporter-tagged constructs (e.g., NS3-luciferase fusion) could allow quantitative, time-resolved measurement of intracellular replication.

Response: A POWV replicon has been reported that we imported that from China (PMID: 38411112) in an attempt to experimentally resolve questions of what stages in virus replication are targeted by TRIM5α. Troubleshooting the clone has taken most of the time spent in revision. However, we could not get this replicon to work in all cell types needed, and the appropriate controls including a RdRP mutant (GNN) were not available. The original report of the replicon found that it naturally acquired a similar mutation that we demonstrated to confer TRIM5α resistance to POWV (NS3 V560M) we have described here. Therefore, this experiment has been published, and demonstrates that replication of the viral RNA is sensitive to the actions of TRIM5α. To begin to assess this in response to the Reviewer query for this work, we examined POWV positive sense (genomic) RNA amplification in MDDCs at 6, 12 and 24 hpi, which demonstrated no difference in early amplification of WT and TRIM5α-resistant virus at 6 and 12 hpi. Only by 24 hpi was the increase in genome replication of rPOWV NS3:T561M observed. This is consistent with our published data that showed no differences in RNA amplification until later in infection (PMID: 31189110). Together with the study by Song et al., 2024, we think that the mechanism is more complicated than the replicon indicates, and functions of NS3 that couple RNA replication to virion assembly may also be affected. As these experiments are more complicated than originally thought, we think that re-design of replicons, use of VLPs in packaging

experiments, and additional mutations in NS3 will be required to correctly identify the restriction block. The replicon findings of Song et al findings is included in the discussion (lines 338-342).

5. The statement that TRIM5α is “not a primary determinant of neuroinvasion” is plausible but may benefit from more nuanced framing. The absence of NS3 mutations in clinical isolates may reflect purifying selection to avoid excessive innate immune activation, rather than a lack of functional relevance of TRIM5α in the human host.

Response: Thank you for this suggestion. This statement has been modified to better reflect this point, which we agree with (line 459-460).

6. The authors might consider discussing whether POWV could evolve compensatory mutations in other nonstructural proteins (e.g., NS2A, NS5) to mitigate the immunostimulatory consequences of T561M while preserving TRIM5α resistance. This could broaden our understanding of viral adaptation strategies.

Response: Thank you for this suggestion. This is an interesting concept - perhaps the most obvious solution for the virus would be to evolve improved antagonism of RLR signaling as a way to actively shut down most of the IFN response to orthoflaviviruses. However, any discussion would be purely speculative, and therefore we would prefer to not include it in the manuscript.

Reviewer #2 (Remarks to the Author):

The authors report TRIM5α inhibits Powassan virus replication by degrading the nonstructural proteins NS2B and NS3 encoding the viral protease and RNA helicase and a mutation in NS3 play a role in this process. The objective of this research is interesting, and the experimental design of the research work is reasonable.

Response: We thank the Reviewer for their positive comments.

1.Please provide the full name of the proper noun when it first appears.

Response: Thank you for this suggestion. We have reviewed the text and edited to include full names of any acronyms used where possible.

2.Please divide Table 1 into three tables based on different viruses according to the characteristics of the table.
Response: Table 1 has now been divided into 3 separate tables for the 3 viruses.

3.Please further optimize the language expression according to the needs of the writing.

Response: We have reviewed the text and edited to try to optimize language.

4.Please give a schematic overview of the whole research based on the results.
Response: Thank you for this suggestion. We have included a schematic that summarizes the results as Figure 9.

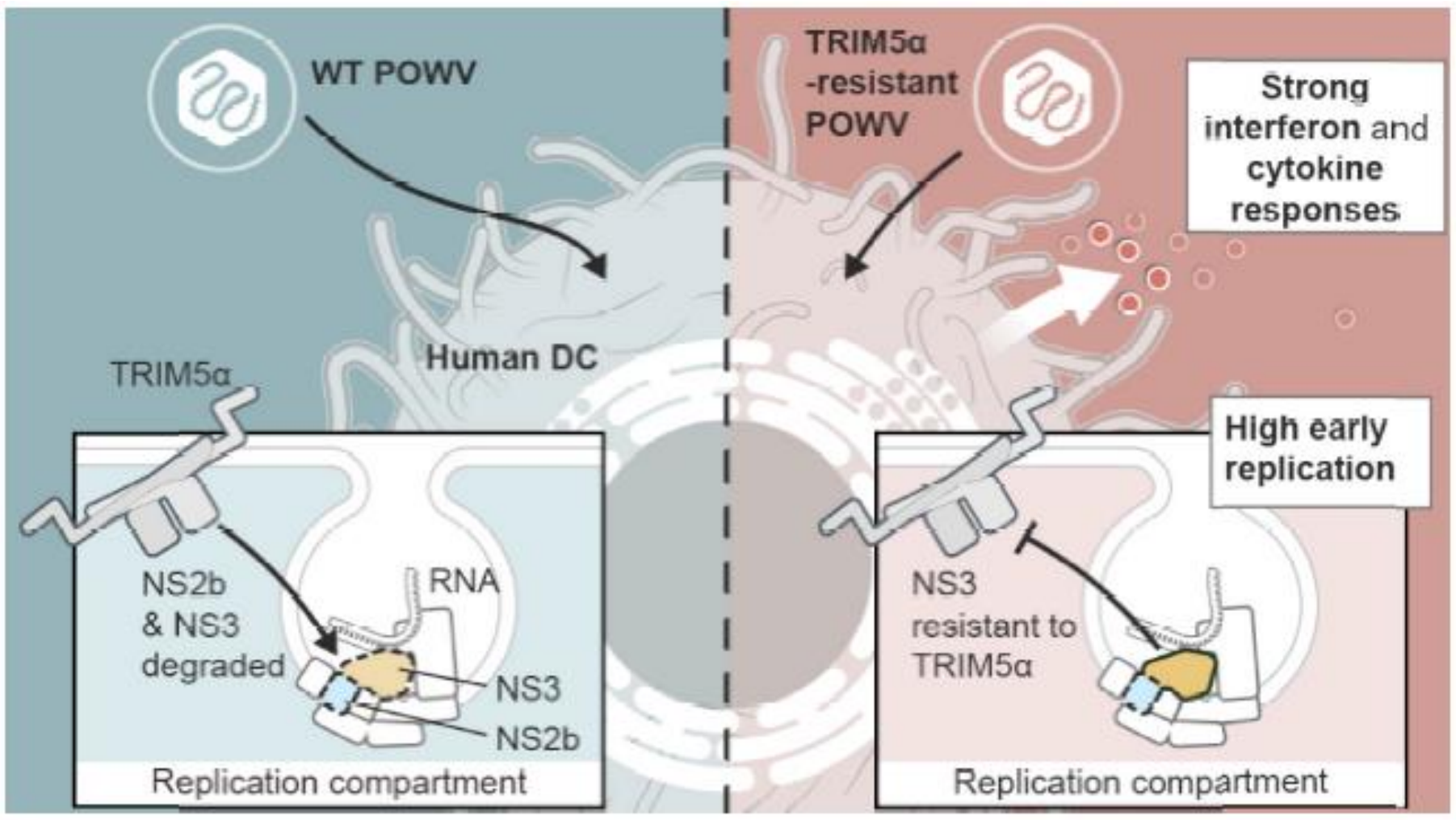

Supplement: Supplementary file 1 [file 42003_2026_10613_MOESM1_ESM.pdf]
