# Supplementary material for: Restraint of Powassan virus replication by TRIM5α facilitates viral avoidance of antiviral immunity
Source: Commun Biol. 2026 Jul 7;9:1419. doi: 10.1038/s42003-026-10613-1 (PMC13720048; doi:10.1038/s42003-026-10613-1)
Supplement: Supplementary file 2 [file 42003_2026_10613_MOESM2_ESM.pdf]

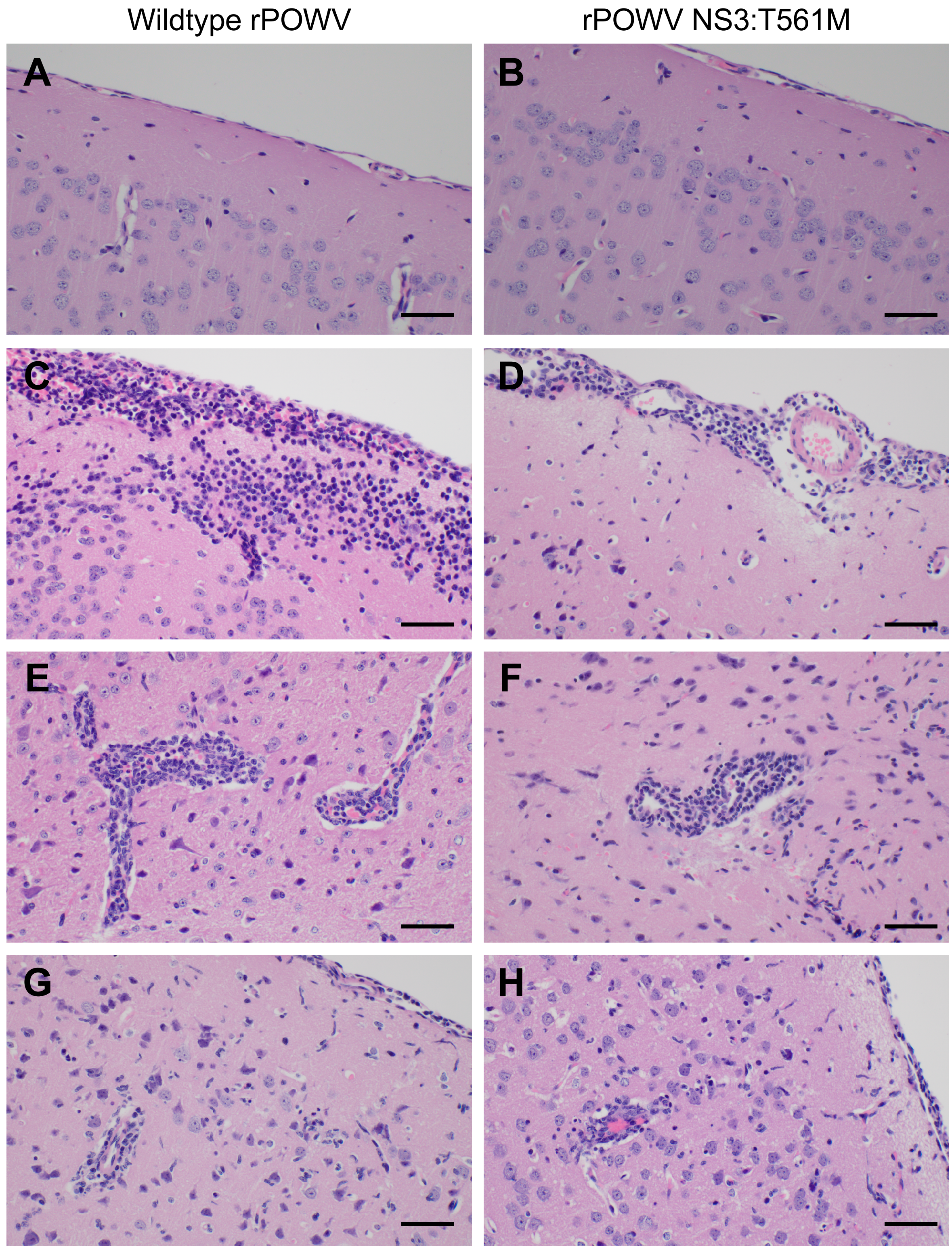

Supplementary Figure 1: Histopathology of POWV infection in the mouse CNS. A, B. At 3 dpi, no inflammation or neuronal necrosis is noted. **C, D.** At 6 dpi, lymphoplasmacytic and neutrophilic inflammation is present within the meninges, and **E, F.** extending into the Virchow-Robbins spaces. **G, H.** At 8 dpi, lymphoplasmacytic and neutrophilic inflammation is present within meninges, Virchow-Robbins spaces, and necrotic cellular debris is present within the neural parenchyma. No differences between WT rPOWV and rPOWV NS3 T561M infections were observed. Scale bar represents 50 μm.

Figure 1A:

TRIM5α-HA

anti-viniculin

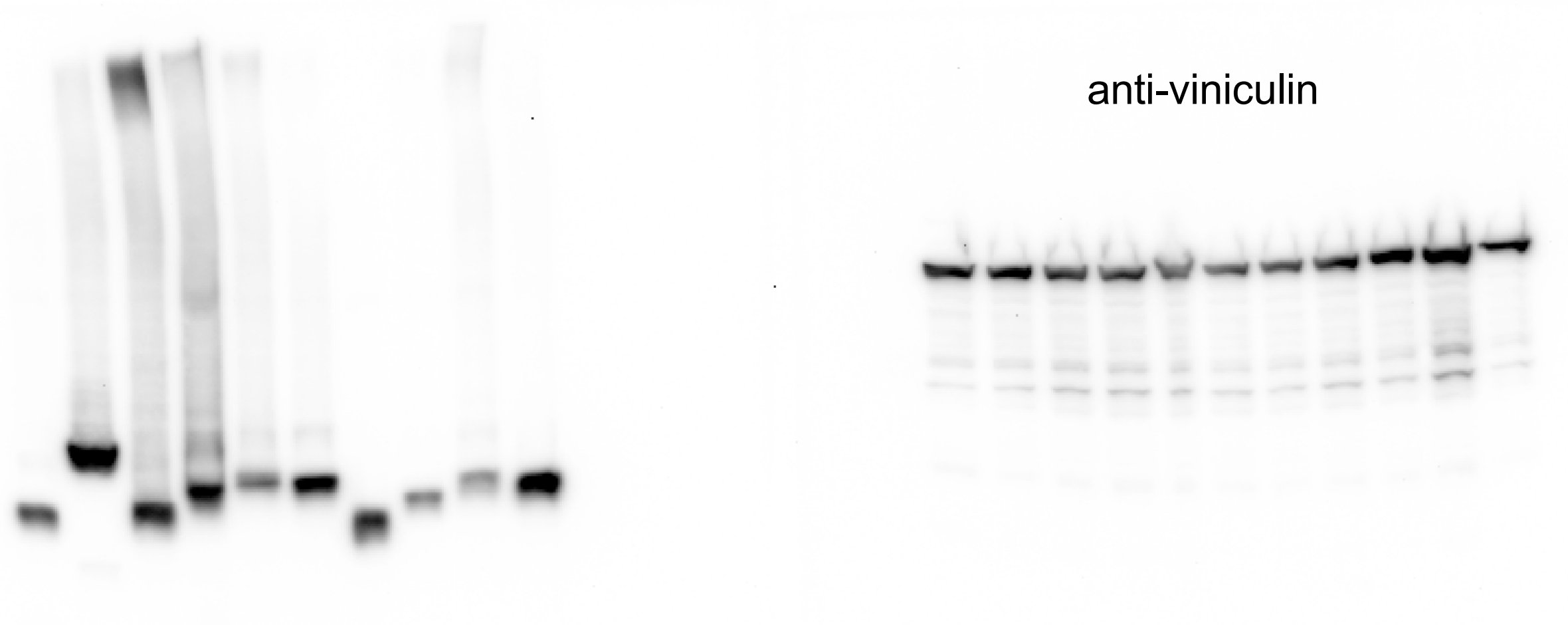

Figure 2H:

WCE: anti-NS3

IP: TRIM5α-HA

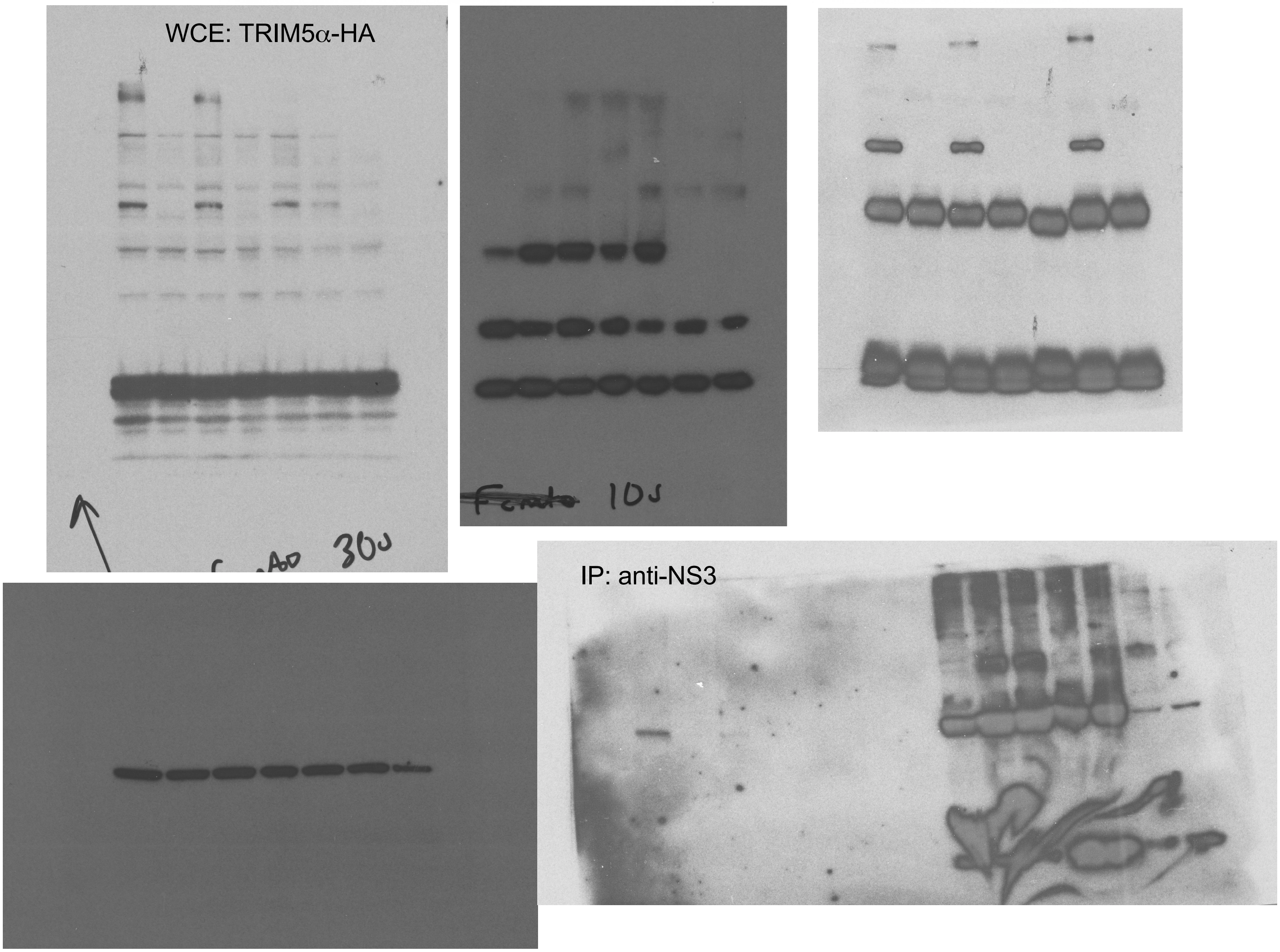

anti-actin

Supplementary Figure 2: Raw Western blots used to generate Figure 1A and Figure 2H.

Figure 6A:

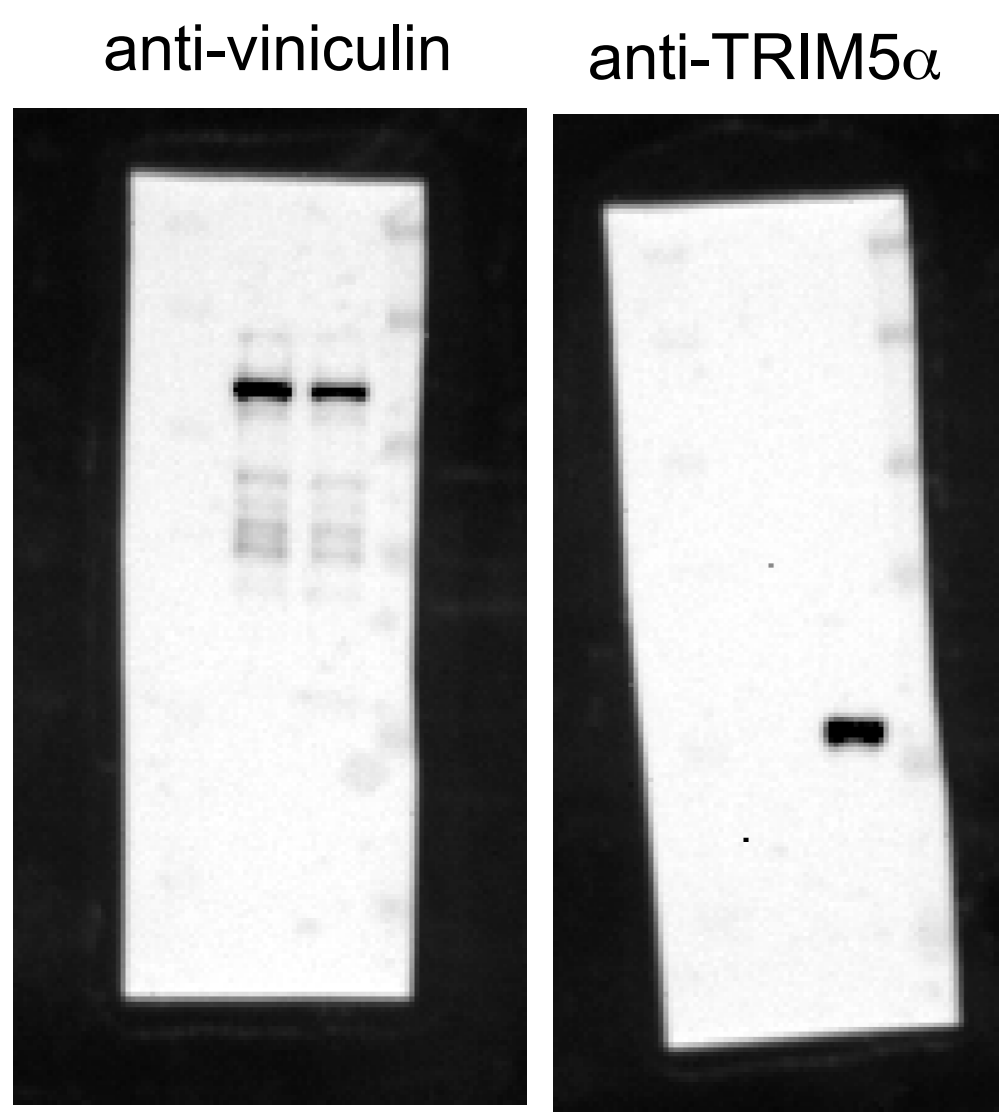

Supplementary Figure 3: Raw Western blots used to generate Figure 6A.

Supplementary Table 1: Primers used to generate LGTV-TP21 molecular clones by CPER

LGTV TP21 CPER primers	Forward Primer (5'-3')	Reverse Primer (5'-3')
Fragment 1	AGATTTTCTTGCGCGTGCATGCGT	CGCTGTCTGTGGGTGCTCTTTTCC
Fragment 2	GGAAAAGAGCACCCACAGACAGCG	CCTTCACTGGAATGCCCATCTCCAAC
Fragment 3	GTTGGAGATGGGCATTCCAGTGAAGG	CAAATGACACTCTTCCCTCGCTGCC
Fragment 4	GGCAGCGAGGGAAGAGTGTCATTTG	ACGGCCCTCTCTAAACATACGGGC
Fragment 5	GCCCGTATGTTTAGAGAGGGCCGT	CAGGGTGTCTCCCTCGGATCC
Fragment 6	GGATCCGAGGGAGACACCCTG	CCATCATCCTCACCAGCTGCACC
Fragment 7	GGTGCAGCTGGTGAGGATGATGG	AGCGGGTGTTTTTCCGAGACACG
Flavilinker	GTGTCTCGGAAAAACACCCGCTGGGTCGGCATGGCATCTCCACC	GCATGCACGCGCAAGAAAATCTCGGTTCACTAAACGAGCTCTGCTTATATAG

Supplementary Table 2: Primers used to generate POWV-LB molecular clones by CPER

POWV LB CPER primers	Forward Primer (5'-3')	Reverse Primer (5'-3')
Fragment 1	AGATTTTCTTGCACGTGTGTGCGGGTGCT	GATCCGACATGGCTTGTCGCTTCCTGT
Fragment 2	ACAGGAAGCGACAAGCCATGTCGGATC	CTTGATTATGAGCAACCCCATGGCCAGTGC
Fragment 3	GCACTGGCCATGGGGTTGCTCATAATCAAG	CTATGGCGCCTCCCTTGGCTATCGATG
Fragment 4	CATCGATAGCCAAGGGAGGCGCCATAG	GTCCAGTCAGGATGTCCACGGCACT
Fragment 5	AGTGCCGTGGACATCCTGACTGGAC	CAAGCTAGTTTGGCACAGCCTCGCGATAC
Fragment 6	GTATCGCGAGGCTGTGCCAAACTAGCTTG	CAGGAAGTACAGCGCCTTTCCGAACCTG

Fragment 7	CAGGTTCGGAAAGGCGCTGTACTTCCTG	AGCGGGTGTTTTTCCGAGTCACACACCA
Flavilinker	GTGACTCGGAAAAACACCCGCTGGGTCGGCATGGCATCTCCACC	GCACACACGTGCAAGAAAATCTCGGTTCACTAAACGAGCTCTGCTTATATAG

Supplementary Table 3: Primers used to generate YFV-H196 molecular clones by CPER

YFV CPER primers	Forward Primer (5'-3')	Reverse Primer (5'-3')
Fragment 1	AGTAAATCCTGTGTGCTAATTGAGGTGC	CCTTTTGGCACCTTCACTTGCATC
Fragment 2	GATGCAAGTGAAGGTGCCAAAAGG	ACTGCGTTCAGGTACTTCCACAGA
Fragment 3	TCTGTGGAAGTACCTGAACGCAGT	GCAGACATGATGCGCATTGTCTTC
Fragment 4	GAAGACAATGCGCATCATGTCTGC	GAGTGCAAGAGCACGCTGATAGTA
Fragment 5	TACTATCAGCGTGCTCTTGCACTC	CAATTTTGCGGTCCCTCTCGAGAC
Fragment 6	GTCTCGAGAGGGACCGCAAAATTG	TCCGCCATCCTAATCAGTTGAACC
Fragment 7	GGTTCAACTGATTAGGATGGCGGA	CCTTTGGATGACAAACACAAAACCACT
Flavilinker	CCTTTGGATGACAAACACAAAACCACTGGGTCGGCATGGCATCTCCACC	GCACCTCAATTAGCACACAGGATTTACTCGGTTCACTAAACGAGCTCTGCTTATATAG
