# Supplementary material for: Restraint of Powassan virus replication by TRIM5α facilitates viral avoidance of antiviral immunity
Source: Commun Biol. 2026 Jul 7;9:1419. doi: 10.1038/s42003-026-10613-1 (PMC13720048; doi:10.1038/s42003-026-10613-1)
Supplement: Supplementary file 3 [file 42003_2026_10613_MOESM3_ESM.pdf]

Description of Additional Supplementary files

File name: Supplementary Data

Description: Raw numerical data from all graphs in the paper
