# Supplementary material for: Restraint of Powassan virus replication by TRIM5α facilitates viral avoidance of antiviral immunity
Source: Commun Biol. 2026 Jul 7;9:1419. doi: 10.1038/s42003-026-10613-1 (PMC13720048; doi:10.1038/s42003-026-10613-1)
Supplement: Supplementary file 4 [file 42003_2026_10613_MOESM5_ESM.pdf]

nature portfolio

Corresponding author(s): Sonja Best

Last updated by author(s): Jun 1, 2026

Reporting Summary

Nature Portfolio wishes to improve the reproducibility of the work that we publish. This form provides structure for consistency and transparency in reporting. For further information on Nature Portfolio policies, see our Editorial Policies and the Editorial Policy Checklist.

Statistics

For all statistical analyses, confirm that the following items are present in the figure legend, table legend, main text, or Methods section.

n/a	Confirmed	
[ ]	[x]	The exact sample size (*n*) for each experimental group/condition, given as a discrete number and unit of measurement
[ ]	[x]	A statement on whether measurements were taken from distinct samples or whether the same sample was measured repeatedly
[ ]	[x]	The statistical test(s) used AND whether they are one- or two-sided<br>*Only common tests should be described solely by name; describe more complex techniques in the Methods section.*
[x]	[ ]	A description of all covariates tested
[x]	[ ]	A description of any assumptions or corrections, such as tests of normality and adjustment for multiple comparisons
[ ]	[x]	A full description of the statistical parameters including central tendency (e.g. means) or other basic estimates (e.g. regression coefficient) AND variation (e.g. standard deviation) or associated estimates of uncertainty (e.g. confidence intervals)
[x]	[ ]	For null hypothesis testing, the test statistic (e.g. *F*, *t*, *r*) with confidence intervals, effect sizes, degrees of freedom and *P* value noted<br>*Give P values as exact values whenever suitable.*
[x]	[ ]	For Bayesian analysis, information on the choice of priors and Markov chain Monte Carlo settings
[x]	[ ]	For hierarchical and complex designs, identification of the appropriate level for tests and full reporting of outcomes
[x]	[ ]	Estimates of effect sizes (e.g. Cohen's *d*, Pearson's *r*), indicating how they were calculated

Our web collection on statistics for biologists contains articles on many of the points above.

Software and code

Policy information about availability of computer code

Data collection	Base calls were demultiplexed and converted into FastQ format using bcl2fastq. Following sequencing, the quality of each sample was assessed using FastQC v0.11.9 (65), Preseq v2.0.3 (66), Picard tools v2.17.1 (67), FastQ Screen v0.9.3 (68), Kraken2 v2.0.8 (69), QualiMap (70), and RSeQC v2.6.4 (71). Sequencing adapters and low-quality bases were trimmed using Cutadapt v1.18 (72). Trimmed reads were aligned to the GENCODE (73) primary human reference genome with STAR v2.7.6a (74). STAR was run in two-pass basic mode to improve discovery and alignment to novel splice junctions. PCR duplicates were marked using Picard's MarkDuplicate utility. Gene expression levels were quantified with RSEM v1.3.3 (75) using GENCODE's v41 comprehensive annotation.
Data analysis	Data analysis was completed using Graph Pad Prism. Lowly expressed genes were filtered from the raw counts matrix using the filterByExpr function from the R package edgeR prior to any downstream analyses. The remaining counts were normalized using TMM scaling factors estimated by the calcNormFactors function within edgeR. Differential gene expression between experimental conditions was tested with the R package limma. Genes considered significantly differentially expressed, via a false-discovery rate (FDR) adjusted p-value ≤ 0.05 and |log2 FC| > 2, were visualized as volcano plot using the R package EnhancedVolcano. Pre-ranked gene set enrichment analysis (GSEA) was performed to assess pathway enrichment using R package clusterProfiler against the msigdbr REACTOME collection. For GSEA, a pre-rank score was calculated using the following formula: -1* (log_(10) (p value)*sign(foldchange)). Pathways or gene sets with a positive normalized enrichment score (NES) are considered up-regulated/activated in the tested group, while pathways or gene sets with negative NES values are considered down-regulated/suppressed relative to the tested group.

For manuscripts utilizing custom algorithms or software that are central to the research but not yet described in published literature, software must be made available to editors and reviewers. We strongly encourage code deposition in a community repository (e.g. GitHub). See the Nature Portfolio guidelines for submitting code & software for further information.

Data

Policy information about availability of data

All manuscripts must include a data availability statement. This statement should provide the following information, where applicable:

- Accession codes, unique identifiers, or web links for publicly available datasets
- A description of any restrictions on data availability
- For clinical datasets or third party data, please ensure that the statement adheres to our policy

All data supporting the findings of this study are available within the paper and its Supplementary Information, with the exception of RNA-Seq data which was deposited into the Gene Expression Omnibus database under accession number GSE325334 and are available at the following URL: https://www.ncbi.nlm.nih.gov/geo/query/acc.cgi?acc=GSE325334.

Research involving human participants, their data, or biological material

Policy information about studies with human participants or human data. See also policy information about sex, gender (identity/presentation), and sexual orientation and race, ethnicity and racism.

Reporting on sex and gender	Human monocyte derived dendritic cells were obtained from the NIH NIH Clinical Center, Department of Transfusion Medicine and deidentified.
Reporting on race, ethnicity, or other socially relevant groupings	N/A
Population characteristics	N/A
Recruitment	Healthy volunteers are recruited into NIH-approved research protocols for collection and distribution of blood components from healthy donors for in vitro research use. Healthy donors over 18 years of age .
Ethics oversight	NIH IRB, NIH Clinical Center Department of Transfusion Medicine. All ethical regulations relevant to human research participants were followed including that informed consent was obtained.

Note that full information on the approval of the study protocol must also be provided in the manuscript.

Field-specific reporting

Please select the one below that is the best fit for your research. If you are not sure, read the appropriate sections before making your selection.

☒ Life sciences ☐ Behavioural & social sciences ☐ Ecological, evolutionary & environmental sciences

For a reference copy of the document with all sections, see nature.com/documents/nr-reporting-summary-flat.pdf

Life sciences study design

All studies must disclose on these points even when the disclosure is negative.

Sample size	Sample sizes for animal studies were determined by power analysis using an 80% confidence interval. Tissue culture sample sizes were generally 3-5, and determined by the nature of the experiment (transformed cell lines vs primary human cells with donor-to-donor variation).
Data exclusions	None
Replication	All experiments were generally performed a minimum of 3 times, with minor exceptions of testing a new cell line for its appropriate to use in subsequent experiments.
Randomization	not performed
Blinding	RNAseq data was analysed by informaticians who were not part ofthe central study and therefore blind to the expected results. Histopathology was read by a blinded pathologist.

Reporting for specific materials, systems and methods

We require information from authors about some types of materials, experimental systems and methods used in many studies. Here, indicate whether each material, system or method listed is relevant to your study. If you are not sure if a list item applies to your research, read the appropriate section before selecting a response.

Materials & experimental systems

n/a	Involved in the study
☐	☒ Antibodies
☐	☒ Eukaryotic cell lines
☒	☐ Palaeontology and archaeology
☐	☒ Animals and other organisms
☒	☐ Clinical data
☒	☐ Dual use research of concern
☒	☐ Plants

Methods

n/a	Involved in the study
☒	☐ ChIP-seq
☒	☐ Flow cytometry
☒	☐ MRI-based neuroimaging

Antibodies

Antibodies used	anti-HA-peroxidase antibody (Roche clone 3F10, #12013819001). HA-tagged constructs for indirect immunofluorescence were detected using anti-HA (Zymed, #71-5500). β-actin was also detected as a loading control using a 1:10,000 dilution of mouse anti-β-actin (Sigma, A5441). A 1:3,000 dilution of goat anti-mouse (Dako, #P0447), anti-rabbit (Thermo Scientific, #P0448) or anti-chicken (Millipore, #12-341) horseradish peroxidase-conjugated antibody was used as a secondary probe. Blots were developed using the ECL Plus detection reagent (GE Healthcare, #RPN2132). Antibodies to detect viral antigens, LGTV (NS3) (previously described in Taylor et al., 2011), and dsRNA antibody J2 (English& Scientific Consulting, #10010200), anti-DYKDDDDK rabbit mAb (D6W5B; Cell Signaling Technologies).
Validation	Generally raised against epitope tags, or previously used extensively in the literature.

Eukaryotic cell lines

Policy information about cell lines and Sex and Gender in Research

Cell line source(s)	Vero E6 cells (ATCC CCL-81), HEK293 cells (ATCC CRL-1573) , BHK-21 cells (ATCC CCL-10) and Hap1 (Horizon Discovery)
Authentication	Cell lines were purchased directly from commercial sources for this work.
Mycoplasma contamination	Cell lines were routinely tested for mycoplasma contamination using PCR based assays.
Commonly misidentified lines (See ICLAC register)	None used

Animals and other research organisms

Policy information about studies involving animals; ARRIVE guidelines recommended for reporting animal research, and Sex and Gender in Research

Laboratory animals	C57BL/6J (6 weeks old) mice (The Jackson Laboratories), equal M and F
Wild animals	None
Reporting on sex	Equal M and F were used.
Field-collected samples	None
Ethics oversight	Animal study protocols were reviewed and approved by the Institutional Animal Care and Use Committee (IACUC) at Rocky Mountain Laboratories (RML), NIAID, NIH in accordance with the recommendations in the Guide for the Care and Use of Laboratory Animals of the NIH.

Note that full information on the approval of the study protocol must also be provided in the manuscript.

Plants

Seed stocks	None
Novel plant genotypes	None
Authentication	N/A
